# Identification and synthesis of decision-making factors in vestibular schwannoma treatment

**DOI:** 10.1007/s00405-026-10104-3

**Published:** 2026-03-15

**Authors:** Lien Segers, Daniele Borsetto, Elke Loos, Steven De Vleeschouwer, Jean-François Daisne, Nicolas Verhaert, Steven W. Mes

**Affiliations:** 1 Department of Otorhinolaryngology, Head and Neck Surgery, University Hospitals Leuven, B3000 Louvain, Belgium; 2 Department of Skull Base Surgery, Cambridge University Hospital, Cambridge, England; 3 KU Leuven, Department of Neurosciences, Research Group Experimental Oto-Rhino-Laryngology, B3000 Louvain, Belgium; 4 Dept. Neuroscience, Leuven Brain Institute, KU Leuven, 3000 Leuven, Belgium; 5 Dept Neurosurgery, University Hospitals Leuven, Leuven, Belgium; 6 Dept Neurosciences, Research Group Experimental Neurosurgery and Neuroanatomy, KU Leuven, Leuven, Belgium; 7 Department of Radiation Oncology, University Hospital Leuven, Louvain, Belgium; 8 Department of Radiation Oncology, Iridium Cancer Network, Wilrijk, Belgium; 9 Department of Otorhinolaryngology and Head and Neck Surgery, Erasmus MC, University Medical Center Rotterdam, Rotterdam, The Netherlands

**Keywords:** Shared decision-making, Vestibular schwannoma, Wait-and-scan, Stereotactic radiotherapy, Microsurgical resection

## Abstract

**Background:**

Managing vestibular schwannomas (VS) involves complex choices among wait-and-scan, stereotactic radiotherapy, and surgery. Although shared decision-making (SDM) is increasingly emphasized, the factors reported to influence treatment choices remain incompletely characterized.

**Objective:**

This systematic review aims to synthesize reported factors influencing treatment decision-making in VS and inform SDM practices.

**Methods:**

In accordance with the PRISMA 2020 guidelines, a systematic literature search was conducted on February 24, 2024, across PubMed, Embase, CINAHL, and Web of Science. Twelve studies were included, comprising patient and/or physician surveys and retrospective chart reviews. Extracted data included patient characteristics, tumor features, physician-related factors, and treatment-related considerations. Levels of evidence were classified using the Oxford Centre for Evidence-based Medicine framework. Most studies were level 4–5, reflecting a predominantly observational and descriptive evidence base.

**Results:**

Tumor size, hearing status, and patient age were frequently reported in association with treatment selection. Psychological factors, including anxiety, reassurance-seeking, and quality-of-life considerations were commonly described in relation to decision-making. Physician factors, such as specialty and communication style, were also reported as relevant. Several studies noted higher satisfaction when patients described active involvement in decision-making and multidisciplinary input.

**Conclusion:**

Decision-making in VS management is multifactorial and highly individualized. Greater emphasis on transparent communication and structured SDM tools may help align treatment decisions with patient preferences. These findings highlight the need for future research focusing on standardized decision aids and the integration of patient-reported outcomes into longitudinal care.

## Introduction

Vestibular schwannomas (VS) are benign tumors originating from Schwann cells of the vestibular nerve [1]. They account for approximately 8% of intracranial tumors in adults and 90% of neoplasms located in the cerebellopontine angle (CPA). VS are the third most common benign intracranial tumor after meningiomas and adenomas of the pituitary gland. They may display continuous growth or reach a certain size before stabilizing or even regressing [2]. As the tumor enlarges within the CPA, it can compress the brainstem or cerebellum and obstruct the fourth ventricle, potentially leading to hydrocephalus and raised intracranial pressure. Improved access to high-resolution MRI has increased the detection of small, asymptomatic VS [3]. However, clinical presentation varies widely, and symptoms are often not proportional to tumor size [4].

The main management options of VS include the wait-and-scan approach (WS), stereotactic radiotherapy (SRT), and microsurgical resection (MSR). WS entails regular imaging to monitor tumor growth and is commonly recommended for asymptomatic patients or tumors without significant progression [5]. Although it minimizes immediate treatment-related risks, WS requires careful long-term follow-up, which may be challenging for some patients. SRT delivers precisely targeted high radiation dose to the tumor to arrest growth while minimizing the dose to surrounding structures [6]. It is frequently selected for small to medium-sized tumors (Koos I to III) displaying growth or in patients unfit for surgery. MSR entails the removal of VS through surgical intervention, generally considered for larger or symptomatic tumors [7]. The three primary surgical approaches, middle cranial fossa, retrosigmoid, and translabyrinthine, have been extensively studied with respect to postoperative cranial nerve preservation. Each approach has distinct advantages and disadvantages that must be weighed in the context of individual patient and tumor characteristics [8].

The European Association of Neuro-Oncology has released guidelines addressing several management approaches, including WS, MSR, and SRT [9]. However, the evidence underpinning these recommendations remains limited. Most cited data correspond to Oxford Centre for Evidence-based Medicine Level III or IV. This reflects both the lack of prospective studies and the complexity of factors influencing decision-making, which are only briefly addressed in the guidelines. Moreover, most studies evaluating long-term outcomes report follow-up periods of less than ten years [10].

Many studies have examined decision-making factors among patients and clinicians, often within relatively narrow clinical contexts [11]. These evaluations typically focus on single elements such as tumor features, symptom severity, patient preferences, or treatment advances, without integrating them into a cohesive, evidence-based framework [12]. As a result, insight into how these factors interact during clinical decision-making remains limited. Therefore, this review aims to synthesize existing evidence to identify and describe key decision-making factors reported across studies.

## Methods

### Search strategy

A systematic review was conducted to identify studies that investigate and report on decision-making processes in the management of VS, incorporating both physician and patient perspectives. The review adhered to the Preferred Reporting Items for Systematic Reviews and Meta-Analyses (PRISMA) 2020 guidelines [13]. The protocol was prospectively registered in the International Prospective Register of Systematic Reviews (PROSPERO; Registration ID: CRD42024607667).

Our literature search was performed across four databases: PubMed, Embase, CINAHL, and Web of Science. The search strategy incorporated terms related to VS and treatment decision-making and was adapted to each database's specific indexing systems. In addition, reference lists of systematic reviews published in the past 10 years were screened to identify potentially relevant studies. The full search strategy, including all search terms and database-specific syntax, is provided in Appendix 1.

The search was conducted without restrictions on publication date. Only articles in English were included. Studies published after February 24, 2024, were not considered. Duplicates were removed prior to screening using Rayyan.ai (https://www.rayyan.ai/). Titles and abstracts were independently screened by two reviewers (LS and SM) against predefined inclusion and exclusion criteria to identify eligible studies (Table 1). Any discrepancies were resolved through consensus discussion. Subsequently, full-text articles of potentially eligible studies were assessed for inclusion using the same criteria.

**Table 1:** Inclusion and exclusion criteria

Category	Inclusion criteria	Exclusion criteria
Participants	Patients with sporadic VS	Patients with other types of schwannomas or other conditions (neurofibromatosis)
	Physicians directly involved in treatment and decision-making	
Outcomes	Studies reporting decision-making factors for treatment options: wait-and-scan approach, radiotherapy, or microsurgical resection	Studies on alternative or adjunctive treatments outside of these main management strategies
	Studies reporting decision-making factors, including psychological, social and demographic influences	Studies focusing on technical aspects or complications unrelated to decision-making
Study design	Qualitative studies, mixed-methods studies, systematic reviews and meta-analyses relevant to decision-making factors	Case reports, editorials, letters to the editor, and narrative reviews
Publication type	Peer-reviewed journal articles and conference papers providing sufficient data for analysis	Non-peer reviewed articles, editorials or papers lacking a full abstract Articles published in languages other than English

### Data extraction

Data extraction was divided into three main areas: study characteristics, decision-making factors, and the qualitative or quantitative nature of how these factors were reported.

Study characteristics included key details such as the author(s), publication year, and country of origin. Study design was recorded, and articles were categorized as randomized controlled trials (RCTs), cohort studies, cross-sectional studies, case series, or qualitative analyses. Sample size and clinical setting, such as tertiary care centers or specialized clinics, were also documented. In addition, the stated study aims and primary outcomes were extracted.

The second area focused on factors influencing decision-making. To structure the analysis, findings from the included articles were grouped into three broad categories: tumor-related, patient-related, and healthcare provider-related aspects. These categories were not predefined but emerged from the literature. Within these categories, studies reported a range of factors, including tumor characteristics (such as size, location and growth), patient aspects (such as age, comorbidities, quality of life and personal preferences), and healthcare provider-related factors (such as surgeon experience, institutional protocols, and multidisciplinary collaboration). The frequency with which factors were reported across studies reflects how often they were examined or mentioned, rather than their relative importance or causal influence on decision-making.

Thirdly, the methods by which decision-making factors were reported and analyzed were examined. Specifically, we assessed whether studies used qualitative methods or a descriptive statistical approach. Factors described as influential based on descriptive analysis were noted separately from those reported as statistically significant through formal testing (i.e., with a reported p-value below a predefined threshold). A formal meta-analysis was not feasible because of substantial heterogeneity in study designs, outcome measures, and analytical methods. Consequently, reported statistical significance reflects within-study analyses and cannot be compared across studies.

Lastly, to characterize the level of evidence and potential limitations of the included studies, we applied the Oxford Centre for Evidence-Based Medicine Levels of Evidence. Most of the 12 included studies were classified as level 4, reflecting their observational and descriptive design. Table 3 provides summarizes the levels of evidence assigned to each study.

## Results

**Fig. 1:**
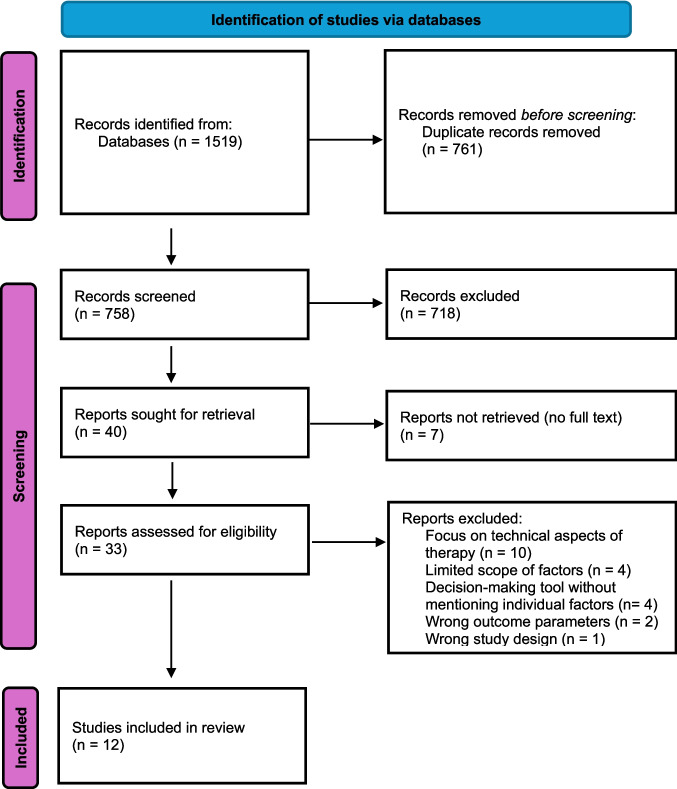
Flow diagram of the literature search and study selection process for this systematic review, in accordance with PRISMA 2020 guidelines

After title and abstract screening, 40 articles were selected for full-text review, of which the complete text could be retrieved for 33 (Fig. 1). Following full-text screening, 12 of these 33 articles were included in the review. Excluded articles were grouped into five categories based on the primary reason for exclusion (Fig. 1). The included studies employed diverse methodologies, including retrospective chart reviews, surveys, and qualitative analyses (Table 2). The Oxford levels of evidence were used to classify the level of evidence of the included studies (Table 3). Sample sizes ranged from 36 specialists involved in VS care to 836 patients with sporadic VS. Across studies, reported outcomes focused on patient- and physician-related factors relevant to decision-making in VS management. Study scope varied, with some addressing patient-reported outcomes and treatment satisfaction, and others examining physician practices and decision-making frameworks.

**Table 2:** Information about the included articles

First author	Country	Year	Type of study	Patient/physician group	Sample size	Main aim	Main outcome measure	Setting
Pogodzinski M. et al	USA	2004	Retrospective chart review	Patients newly diagnosed with VS from January 2000 through December 2001	N = 139	To examine the initial treatment choices made by patients with VS	The proportion of patients selecting wait-and-scan, stereotactic radiotherapy or microsurgical resection, and the factors influencing their choice	Tertiary care medical center
Müller S. et al	Germany	2007	Postal questionnaire survey (cross-sectional)	Patients with VS in Germany	N = 739	To investigate the decision-making process of patients with VS	Information provided to patients about treatment options, influence of medical counseling and external factors on treatment choice, and patient satisfaction with decision-making	Postal patient survey
Nellis J. et al	USA	2016	Prospective observational study	Patients with unilateral VS presenting for surgical evaluation	N = 216	To identify factors influencing the decision to undergo surgical resection versus active surveillance in patients with VS	The association of demographic, clinical, tumor-associated, and psychological factors with the decision to pursue surgery, analyzed through univariate and multiple logistic regression	Tertiary care medical center
Moshtaghi O. et al	USA	2017	Cross-sectional survey	Patients diagnosed with VS who are members of the Acoustic Neuroma Association (ANA)	N = 789	To assess the decision-making process of patients with VS, including factors influencing treatment choices and satisfaction with their decisions	Factors influencing patient decision-making, time spent with physicians, second opinions sought, patient satisfaction with treatment decisions​	Online patient survey
Carlson M.J. et al	USA	2018	Cross-sectional survey	Patients with small- to medium-sized sporadic VS who underwent SRT, MSR or WS	N = 539	To assess patient motivation behind treatment selection and long-term satisfaction with their chosen treatment	Primary motivation for treatment choice, satisfaction rates with treatment decision, and willingness to recommend the same treatment to others	Online patient survey
Hentschel M. et al	Netherlands	2018	Cross-sectional survey	Otolaryngologists from 11 different countries specializing in VS management	N = 36	To compare international diagnostic and management strategies for VS and identify variations in clinical practice	Differences in guideline use, diagnostic criteria, management preferences, and factors influencing treatment decisions​	Online doctor survey targeting ENT specialists
Goshtasbi K. et al	USA	2020	Cross-sectional survey	Patients diagnosed with VS responding to a survey from January to March 2017	N = 789	To assess the current state of VS diagnosis and management, trends in treatment choices, and the role of various specialists in influencing treatment plans	Trends in surgical vs. non-surgical management, specialist influence on treatment decisions, and patient satisfaction with treatment choices	Online patient survey
Kleijwegt M. et al	Netherlands	2020	Retrospective chart review	Patients with VS who were initially managed conservatively at Leiden University Medical Center	N = 836	To identify clinical and tumor characteristics that predict a change from conservative management to active treatment in VS patients	Factors influencing the decision to switch from conservative management	Tertiary care medical center
Macielak R.J. et al	USA	2020	Cross-sectional survey	Clinicians who specialize in the management of VS	N = 110	To assess practice patterns among providers managing VS	Responses to questions on the management and anticipated outcomes of VS for a series of common clinical scenarios	Online doctor survey targeting specialists in VS care
Neve O.M. et al	Netherlands	2020	Qualitative study (inductive thematic analysis)	Patients with small- to medium-sized VS	N = 18	To identify patient-reported factors that influence treatment decisions for VS	Themes influencing treatment choices, categorized as medical factors and patient-related factors	Tertiary care medical centers: face-to-face patient interviews
Harvey E. et al	USA	2022	Retrospective chart review	Patients diagnosed with VS between 2009 and 2019	N = 197	To identify demographic and clinical features impacting the initial treatment pathway for VS	Correlation of demographic and clinical factors with initial treatment pathway using statistical analyses (χ2 test, ANOVA, and multivariate logistic regression)	Tertiary care medical center
Pruijn I. et al	Netherlands	2023	Cross-sectional qualitative study, using a phenomenological approach	Patients with unilateral VS, contacted through the Dutch VS association Stichting Hoormij	N = 231	To identify patient-preferred treatment outcomes and explore patient-reported symptoms and management-related side effects of VS and their impact on health-related quality of life (HRQoL)	Patient-preferred outcomes, ranked symptoms and side effects, their impact on HRQoL, and frequency of occurrence, analyzed through qualitative content analysis	Online patient survey

**Table 3:** Rating of evidence of each study (Oxford Centre for Evidence-Based Medicine Levels of Evidence)

Author	Level of evidence	Reason
Pogodzinski M. et al	Level 4	Retrospective chart review. Usage of descriptive statistics without strong comparative evidence
Müller S. et al	Level 4	Cross-sectional postal questionnaire survey. Usage of descriptive statistics without strong comparative evidence
Nellis J. et al	Level 3	Prospective cohort study. No randomized control
Moshtaghi O. et al	Level 4	Cross-sectional survey. Usage of descriptive statistics without strong comparative evidence
Carlson M.J. et al	Level 4	Cross-sectional survey. Usage of descriptive statistics without strong comparative evidence
Hentschel M. et al	Level 4	Cross-sectional survey. Usage of descriptive statistics without strong comparative evidence
Goshtasbi K. et al	Level 4	Cross-sectional survey. Usage of descriptive statistics without strong comparative evidence
Kleijwegt M. et al	Level 4	Retrospective chart review. Usage of descriptive statistics without strong comparative evidence
Macielak R.J. et al	Level 4	Cross-sectional survey. Usage of descriptive statistics without strong comparative evidence
Neve O.M. et al	Level 5	Qualitative study (inductive thematic analysis)
Harvey E. et al	Level 4	Retrospective chart review. Usage of descriptive statistics without strong comparative evidence
Pruijn I. et al	Level 5	Qualitative research without systematic analysis

### Patient and tumor factors

**Fig. 2:**
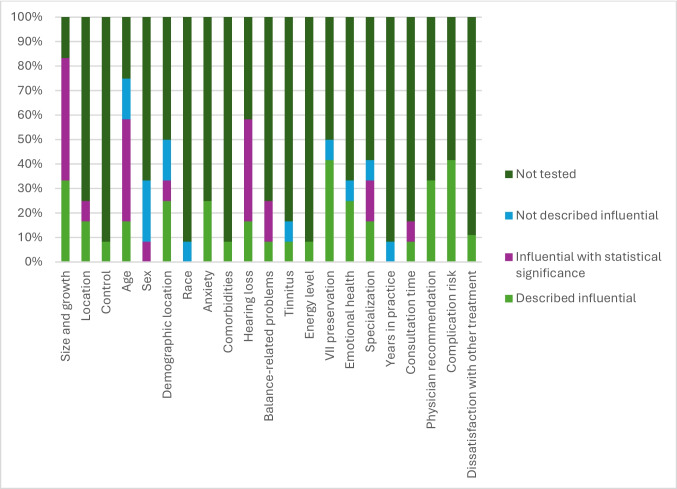
Decision-making factors and their reported statistical significance across included studies. This figure illustrates the proportion of studies that reported each decision-making factor as statistically significant, described it as influential without formal statistical testing, reported it as statistically non-significant, or did not report it. Factors are grouped into tumor factors (*size and growth, location, tumor control*), patient-related factors (*age, sex, demographic location, race, anxiety, comorbidities*), tumor-related symptoms (*hearing loss, balance-related problems, tinnitus, energy level, facial nerve preservation, emotional health*), physician-related factors (*specialization, years in practice, consultation time, physician recommendation*), and treatment-related factors (*perceived complication risk, dissatisfaction with other treatment*). Reported statistical significance reflects within-study analyses only and should not be interpreted as indicating relative importance, effect size or strength of evidence across factors or studies

All twelve studies reported on patient-specific and tumor-related characteristics in relation to decision-making(Supplemental Table 1, Fig. 2). Tumor size and growth were reported as statistically significant within six individual studies and described as influential in four, with larger or growing tumors commonly associated with a shift from conservative management to active treatment [14–23]. Reported size thresholds for surgery were generally around a maximum tumor diameter of 30 mm [15–17, 23]. However, four studies reported a threshold of approximately 20 mm for treatment with MSR or SRT, particularly in symptomatic patients [14, 15, 18, 23]. Tumor location was less frequently examined, but extracanalicular location was described as influential in two studies [18, 19]. One study reported extracanalicular tumor location as statistically significant, in association with a change in initial treatment strategy and subsequent intervention [14].

Patient age was frequently examined as a potential factor in decision-making, with older age more often associated with conservative management rather than surgical intervention, reflecting perceived surgical risks and potential impact on quality of life. Two studies reported a cut-off of 65 years, above which patients were less likely to undergo active treatment [14, 16]. Two retrospective chart reviews [14, 15], reflecting combined patient and physician perspectives, identified age as associated with treatment choice.

Demographic variables, such as sex, race, and geographic location, showed inconsistent associations with decision making, with some studies reporting statistical significance and others finding no clear relationship. One study reported that patients living in rural areas had limited access to specialized centers, which may influence treatment choices toward more conservative approaches because of practical constraints [15]. Three articles noted that patients living in countries such as Germany, France, and the USA were more likely to undergo MSR than in other countries, suggesting international variation in VS management practices [17, 18, 24]. Comorbidities were infrequently examined. However, one study described them as a potential influence on treatment decisions [16].

### Symptoms and quality of life

From the patient perspective, hearing loss, balance-related issues, and tinnitus, were the most frequently reported symptoms in relation to decision-making. Hearing loss was reported as statistically significant in multiple studies and was frequently described in association with decisions to pursue active intervention [14–17, 20, 22, 25]. Two studies reported that patients with severe hearing loss were more likely to undergo MSR [20, 22]. In contrast, another study found that these patients more often underwent WS than patients with intact hearing, for whom hearing preservation was described as a primary management goal [16].

Balance issues and tinnitus were less frequently examined but were reported as concerns in some patient groups, particularly among individuals experiencing dizziness or instability. Emotional health and QoL considerations, including concerns to facial nerve preservation, were also commonly reported as relevant to decision-making. One study described energy restoration as an influential consideration [25].

Several studies highlighted patient-reported expectations as relevant in SDM. Patients who prioritized current quality of life more often favored non-invasive options [20], whereas those expressing a preference for definitive tumor removal more frequently opted for surgery [19]. Neve et al. also reported that some patients expressed concerns about tumor location, which was described as a reason for seeking treatment [19].

Five studies addressed psychological and social influences in relation to decision-making in VS [17, 19, 21, 22, 25]. Patients reporting higher levels of anxiety, decisional conflict, or pre-treatment distress more frequently described seeking multiple opinions [19, 21]. Social support networks, including family and peer discussions, were also commonly reported as relevant, with patients describing greater confidence in their decisions when strong family support was present [19]. Participation in patient groups or online forums was associated with greater perceived empowerment and decisional certainty, indicating that peer support may contribute to the decision-making process [17].

In settings where physicians predominantly guided treatment choice by presenting only one therapeutic option, lower levels of patient autonomy were reported, sometimes accompanied by dissatisfaction when outcomes did not align with patient expectations [17]. In contrast, patients who described involvement in more participatory decision-making models, actively weighing risks and benefits, reported higher satisfaction levels [20].

From the physician perspective, symptoms were reported as less influential in decision-making than from the patient perspective [18, 24]. However, preservation of the facial nerve was described as a relevant consideration influencing physicians’ treatment choices [24]. One study briefly noted that symptoms were taken into account but did not specify which symptoms were considered [18].

### Physician-related factors

Physician recommendations were frequently reported by patients as an important determinant in decision-making, with four studies reporting statistical significance for this association [17, 19–21]. Four studies described physician specialization as an influential factor [16, 17, 23, 24], whereas one study reported no such association [15]. One study also examined years in practice and found no significant association with treatment choice [24]. General otolaryngologists and neurotologists were described as more influential than neurosurgeons in one study [23]. In another study, nearly all patients who were seen exclusively by an SRT-physician underwent SRT, whereas patients seen by neurotologists more often chose WS [16]. Time spent with the physician was also reported as relevant, with longer consultations allowing for more extensive discussion of treatment options [17, 21].

One study reported that patients who sought second opinions described higher satisfaction with their treatment choice, while those reporting lower levels of trust in their physician were more likely to seek an additional opinion [19]. Patients receiving conflicting recommendations from different specialists reported greater levels of confusion and decisional fatigue [17, 21, 24].

### Treatment-related factors

Complication risk was frequently reported as a patient concern and was described as influential in five studies [17, 19–21, 25]. In addition, patients reporting dissatisfaction with initial treatment decisions more often described switching management strategies [21]. Variability in practice patterns among clinicians and across international settings was also reported [18].

Treatment satisfaction was also reported in relation to long-term outcomes, with patients experiencing fewer complications and better tumor control describing greater contentment with their treatment choice [20]. This study reported that avoidance of surgical adverse effects was a commonly cited reason for choosing SRT or WS. The two studies with longer follow-up periods suggested that, despite high initial satisfaction, later challenges such as hearing loss and balance problems were associated with less favorable retrospective perceptions of decision-making and lower self-esteem scores [20, 22].

Finally, patients who underwent surgical intervention more often reported higher satisfaction when expectations regarding postoperative outcomes were adequately addressed [20, 24]. Conversely, patients who opted for conservative management sometimes reported regret when tumor progression resulted in subsequent intervention [14]. Participation in structured decision-making programs incorporating risk stratification and outcome simulations was associated with lower levels of decisional regret [19].

## Discussion

### Interpretation of key findings

This systematic review highlights the multifaceted nature of the decision-making in VS management, which is shaped by an interplay of tumor characteristics, patient-reported preferences and concerns, physician practices, and treatment-related considerations.

Although tumor size and growth were consistently reported as relevant across studies [14–23], a difference in perspective emerged. Patients more often emphasized hearing status and symptom burden, including facial nerve function [14–17, 20, 22, 25], whereas clinicians more frequently focused on facial nerve preservation and technical considerations [24]. One study reported that patients with severe hearing loss more often opted for a WS approach compared with those with preserved hearing [16]. This pattern may reflect a perception of reduced urgency for intervention when functional hearing is already compromised, particularly in the context of perceived surgical risks. Conversely, patients with better hearing may consider earlier treatment to preserve function. However, these associations varied across studies and settings, illustrating the balance between potential hearing deterioration during observation and the perceived benefits of early intervention.

Patient-related factors such as age, comorbidities, and psychological aspects were examined in many studies, but showed inconsistent associations with treatment choice. Several studies reported that younger patients or those expressing concern about tumor progression more often favored surgical intervention, potentially reflecting a desire for perceived control or definitive treatment [14–17, 19–23].

The absence of a standardized decision-making framework may contribute to observed variability in treatment choices, with physician specialty and counseling style as influential factors [16]. This suggests that recommendations are shaped not only by clinical characteristics but also by provider-specific preferences [11]. Longer consultation times were commonly associated with more balanced discussions, potentially facilitating patient-centered decision-making [18, 26]. Further exploration of physician-related factors, including risk perception, specialty background and organizational context, may support more consistent counseling practices and improved multidisciplinary alignment. Rather than providing prescriptive guidance, this synthesis may serve as a framework for future research by identifying candidate decision-making factors for more systematic evaluation and quantification in prospective studies.

### Comparison with existing literature

These findings are consistent with literature on decision-making in other skull base tumors. In meningioma management, patients often facor conservative strategies or delayed intervention when tumor growth is slow and symptoms are mild, prioritizing quality of life over aggressive treatment [27]. In contrast, decision-making in pituitary tumors is more strongly driven by specific clinical factors such as visual impairment and hormonal dysfunction, with patient preferences incorporated within a more medically directed framework [28, 29]. Together, these comparisons highlight that while SDM is central in neuro-oncology, its practical application varies by tumor type and clinical context.

Beyond retrospective and cross-sectional studies, the V-REX RCT represents an important advance in the VS evidence base [30]. By prospectively comparing upfront SRT with WS in small- to medium-sized tumors, V-REX demonstrated superior short-term tumor control with early SRT, without significant detriment to hearing-related or overall QoL at two years. These findings may influence future SDM discussions by altering perceptions of the balance between tumor control and functional preservation. However, as most studies included in this review predate V-REX, the decision-making factors identified largely reflect historical practice patterns.

### Limitations

This review has several limitations. Most included studies were retrospective or cross-sectional, introducing potential recall and selection bias [31, 32]. While patient-reported outcomes obtained through surveys and interviews provide valuable insight, they remain subjective and may not capture all clinical considerations underlying treatment decisions [33]. In addition, heterogeneity in the decision-making factors examined across studies limits direct comparison and synthesis.

Another limitation is the predominance of low-level evidence. Using the Oxford Centre for Evidence-Based Medicine framework, most studies were classified as level 4–5, limiting inferential strength. Accordingly, this synthesis should be interpreted as hypothesis-generating, identifying patterns and associations rather than establishing causal relationships [34]. The lack of standardized measurement also precluded formal meta-analysis [35].

All included studies evaluated patients managed before publication of the V-REX trial [30]. As this study provides the first high-level prospective evidence in VS management, its findings may influence both physician recommendations and patient preferences. Consequently, the decision-making factors identified in this review may not fully reflect contemporary or future practice patterns, underscoring the need for updated research in light of emerging evidence.

Finally, few studies examined the clinician perspective in isolation. Only two studies focused exclusively on healthcare providers' decision-making rationale [18, 24], while most presented mixed perspectives without clearly distinguishing physician- and patient-derived factors [14, 15]. This limits insight into professional, institutional, and systemic influences on clinician behavior and represents an important gap in the literature.

### Future research directions

Future research should prioritize prospective, multicenter studies with standardized methodologies to validate key decision-making factors [35]. The development of structured SDM tools, digital education platforms, and tailored decision aids may help align treatment choices with patient expectations and improve long-term satisfaction [36]. Integrating clinical, biological and psychosocial data into personalized care frameworks could further support consistent, patient-centered management [37]. Consensus-based guidelines reflecting the full spectrum of patient needs are needed to reduce international variability [36].

## Conclusion

Management of VS involves a highly individualized and multifactorial decision-making process shaped by tumor characteristics, functional considerations, patient preferences, psychological readiness, and physician-related factors. Given current variability in practice and the predominance of low-level evidence, future efforts should focus on rigorous prospective research and the development of structured, patient-centered decision making frameworks to enable more consistent and evidence-informed care.
